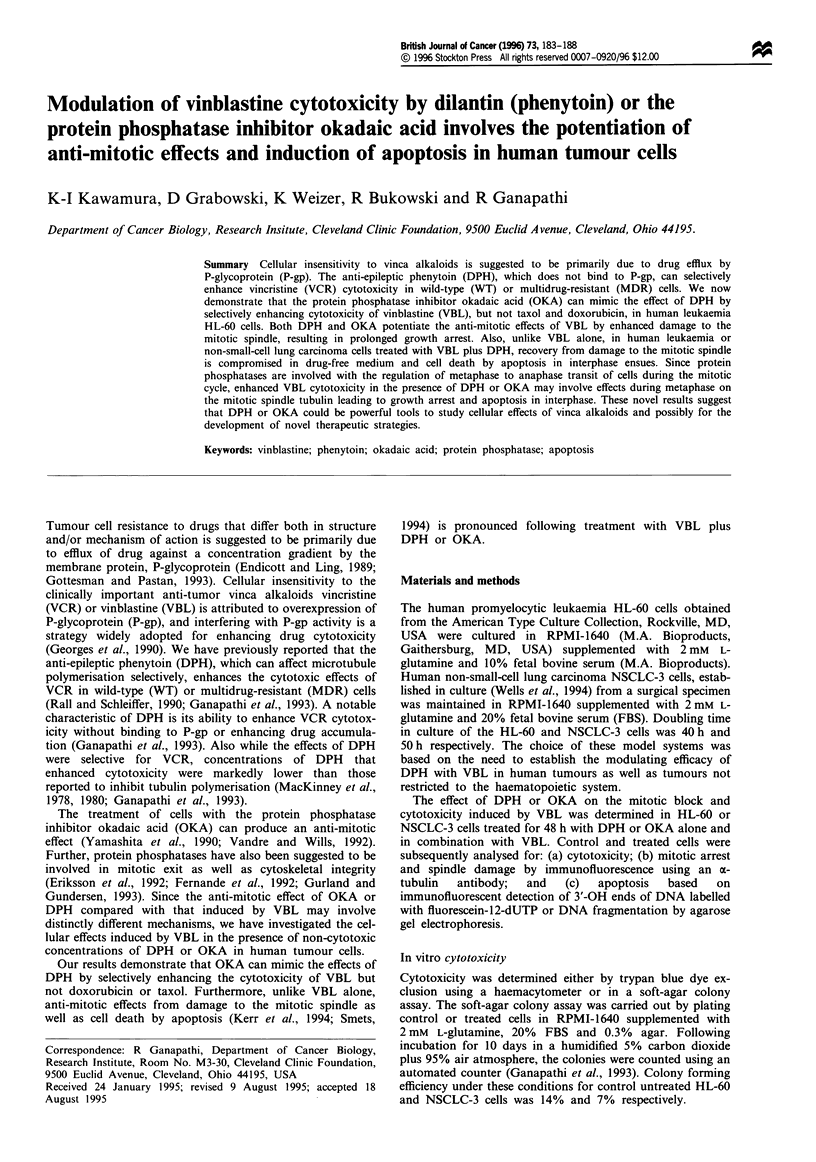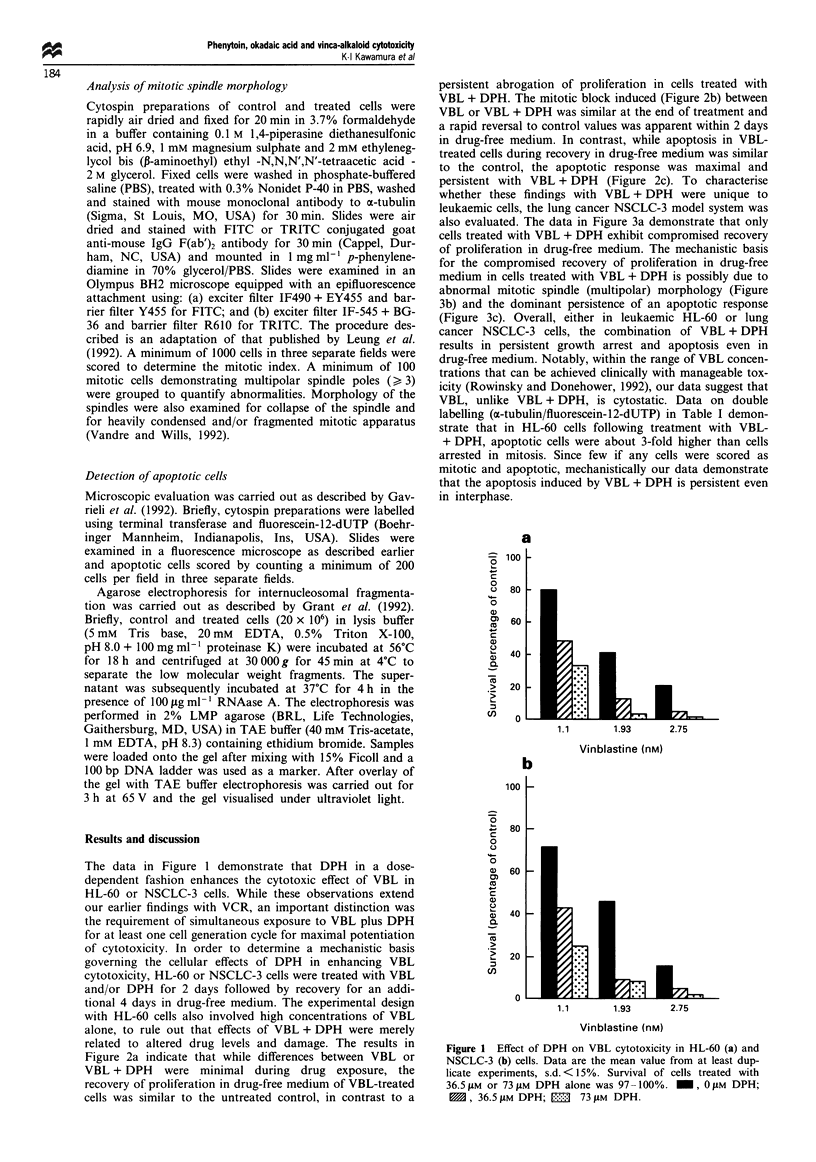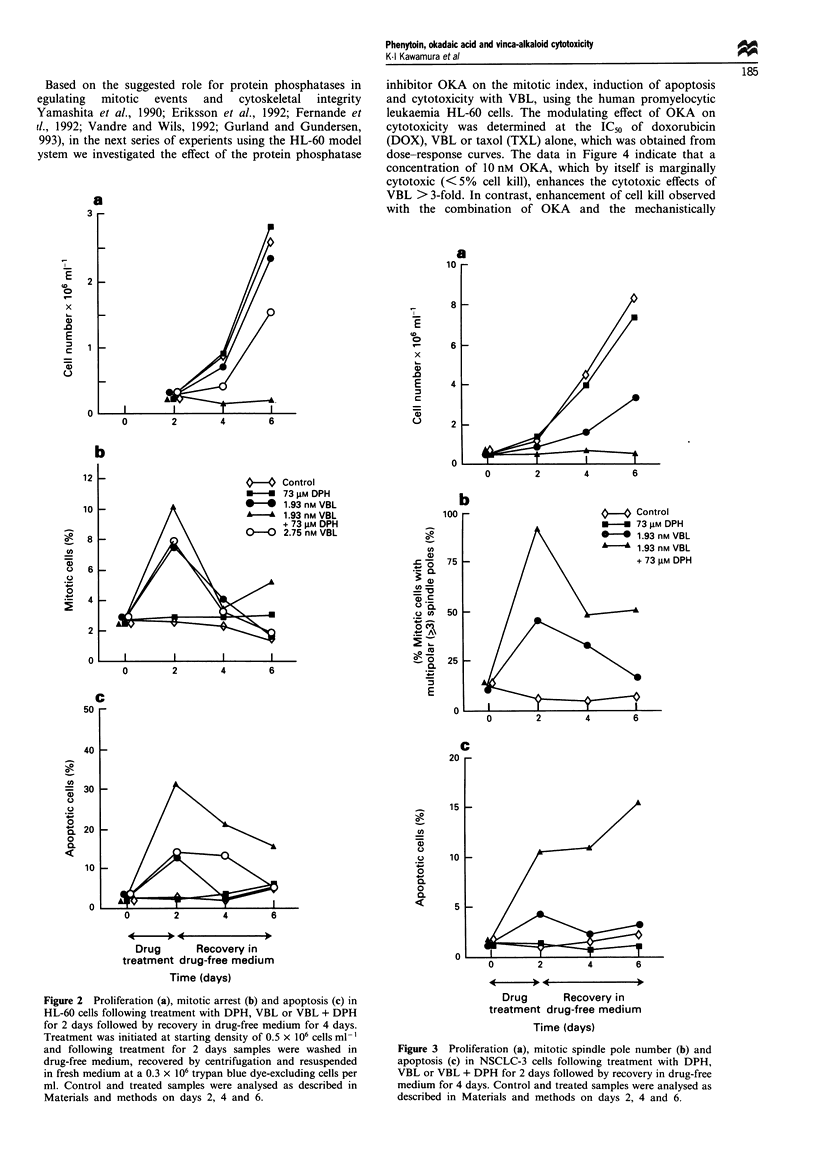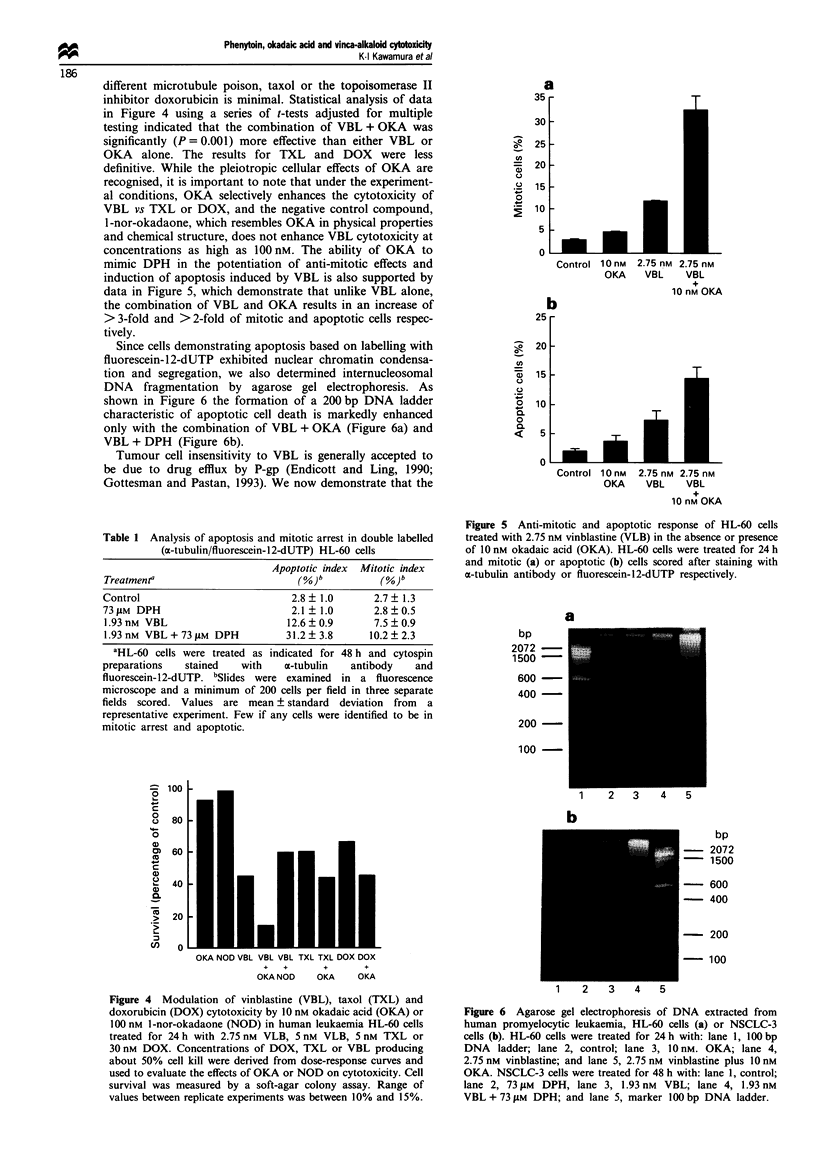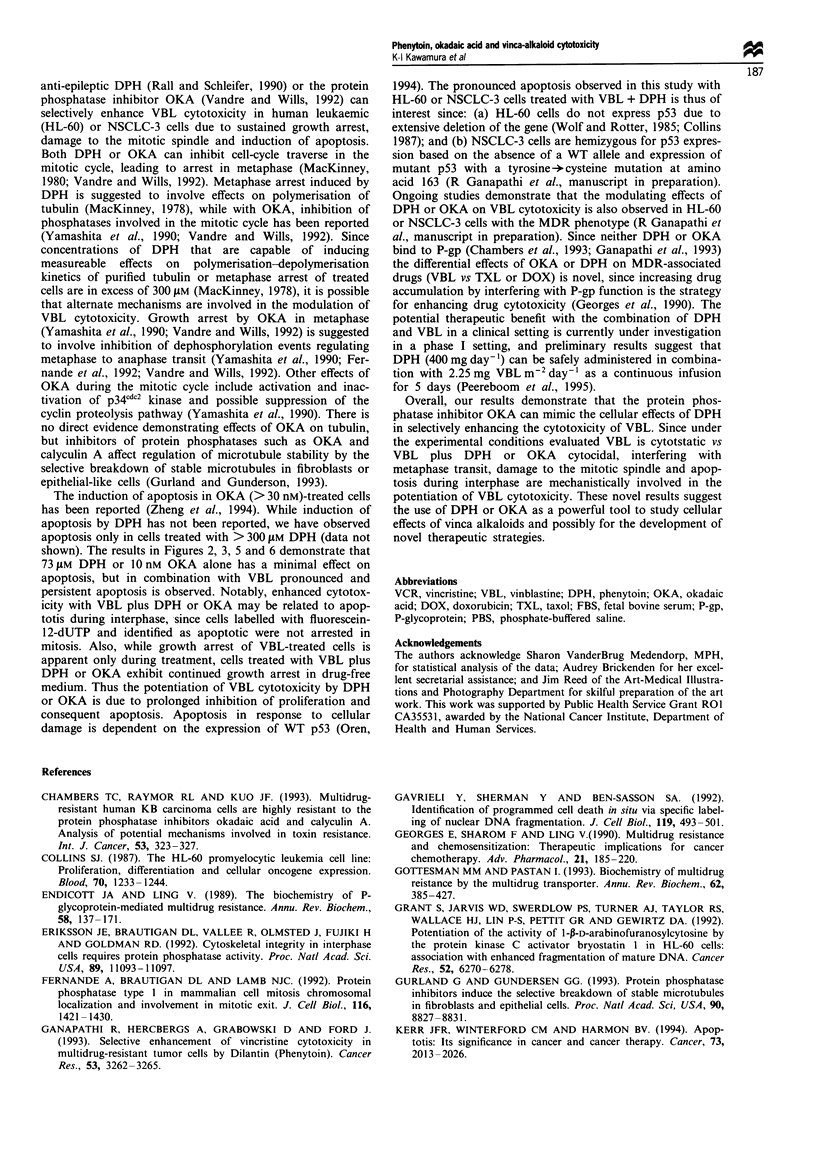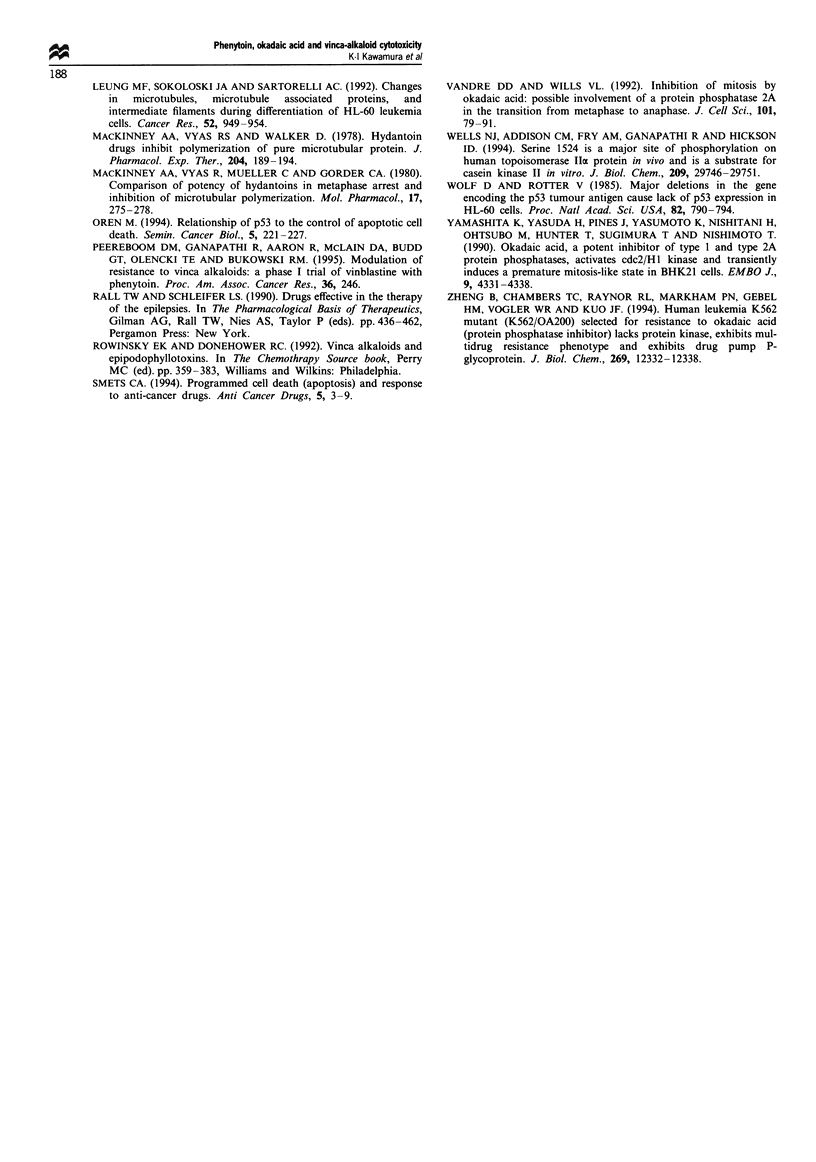# Modulation of vinblastine cytotoxicity by dilantin (phenytoin) or the protein phosphatase inhibitor okadaic acid involves the potentiation of anti-mitotic effects and induction of apoptosis in human tumour cells.

**DOI:** 10.1038/bjc.1996.33

**Published:** 1996-01

**Authors:** K. I. Kawamura, D. Grabowski, K. Weizer, R. Bukowski, R. Ganapathi

**Affiliations:** Department of Cancer Biology, Cleveland Clinic Foundation, Ohio 44195, USA.

## Abstract

**Images:**


British Journal of Cancer (1996) 73, 183-188
© 1996 Stockton Press All rights reserved 0007-0920/96 $12.00

### Modulation of vinblastine cytotoxicity by dilantin (phenytoin) or the protein phosphatase inhibitor okadaic acid involves the potentiation of anti-mitotic effects and induction of apoptosis in human tumour cells

K-I Kawamura, D Grabowski, K Weizer, R Bukowski and R Ganapathi

*Department of Cancer Biology, Research Insitute, Cleveland Clinic Foundation, 9500 Euclid Avenue, Cleveland, Ohio 44195.*

**Summary** Cellular insensitivity to vinca alkaloids is suggested to be primarily due to drug efflux by P-glycoprotein (P-gp). The anti-epileptic phenytoin (DPH), which does not bind to P-gp, can selectively enhance vincristine (VCR) cytotoxicity in wild-type (WT) or multidrug-resistant (MDR) cells. We now demonstrate that the protein phosphatase inhibitor okadaic acid (OKA) can mimic the effect of DPH by selectively enhancing cytotoxicity of vinblastine (VBL), but not taxol and doxorubicin, in human leukaemia HL-60 cells. Both DPH and OKA potentiate the anti-mitotic effects of VBL by enhanced damage to the mitotic spindle, resulting in prolonged growth arrest. Also, unlike VBL alone, in human leukaemia or non-small-cell lung carcinoma cells treated with VBL plus DPH, recovery from damage to the mitotic spindle is compromised in drug-free medium and cell death by apoptosis in interphase ensues. Since protein phosphatases are involved with the regulation of metaphase to anaphase transit of cells during the mitotic cycle, enhanced VBL cytotoxicity in the presence of DPH or OKA may involve effects during metaphase on the mitotic spindle tubulin leading to growth arrest and apoptosis in interphase. These novel results suggest that DPH or OKA could be powerful tools to study cellular effects of vinca alkaloids and possibly for the development of novel therapeutic strategies.

**Keywords:** vinblastine; phenytoin; okadaic acid; protein phosphatase; apoptosis

Tumour cell resistance to drugs that differ both in structure and/or mechanism of action is suggested to be primarily due to efflux of drug against a concentration gradient by the membrane protein, P-glycoprotein (Endicott and Ling, 1989; Gottesman and Pastan, 1993). Cellular insensitivity to the clinically important anti-tumor vinca alkaloids vincristine (VCR) or vinblastine (VBL) is attributed to overexpression of P-glycoprotein (P-gp), and interfering with P-gp activity is a strategy widely adopted for enhancing drug cytotoxicity (Georges *et al.*, 1990). We have previously reported that the anti-epileptic phenytoin (DPH), which can affect microtubule polymerisation selectively, enhances the cytotoxic effects of VCR in wild-type (WT) or multidrug-resistant (MDR) cells (Rall and Schleiffer, 1990; Ganapathi *et al.*, 1993). A notable characteristic of DPH is its ability to enhance VCR cytotoxicity without binding to P-gp or enhancing drug accumulation (Ganapathi *et al.*, 1993). Also while the effects of DPH were selective for VCR, concentrations of DPH that enhanced cytotoxicity were markedly lower than those reported to inhibit tubulin polymerisation (MacKinney *et al.*, 1978, 1980; Ganapathi *et al.*, 1993).

The treatment of cells with the protein phosphatase inhibitor okadaic acid (OKA) can produce an anti-mitotic effect (Yamashita *et al.*, 1990; Vandre and Wills, 1992). Further, protein phosphatases have also been suggested to be involved in mitotic exit as well as cytoskeletal integrity (Eriksson *et al.*, 1992; Fernande *et al.*, 1992; Gurland and Gundersen, 1993). Since the anti-mitotic effect of OKA or DPH compared with that induced by VBL may involve distinctly different mechanisms, we have investigated the cellular effects induced by VBL in the presence of non-cytotoxic concentrations of DPH or OKA in human tumour cells.

Our results demonstrate that OKA can mimic the effects of DPH by selectively enhancing the cytotoxicity of VBL but not doxorubicin or taxol. Furthermore, unlike VBL alone, anti-mitotic effects from damage to the mitotic spindle as well as cell death by apoptosis (Kerr *et al.*, 1994; Smets, 1994) is pronounced following treatment with VBL plus DPH or OKA.

Correspondence: R Ganapathi, Department of Cancer Biology, Research Institute, Room No. M3-30, Cleveland Clinic Foundation, 9500 Euclid Avenue, Cleveland, Ohio 44195, USA
Received 24 January 1995; revised 9 August 1995; accepted 18 August 1995

### Materials and methods

The human promyelocytic leukaemia HL-60 cells obtained from the American Type Culture Collection, Rockville, MD, USA were cultured in RPMI-1640 (M.A. Bioproducts, Gaithersburg, MD, USA) supplemented with 2 mM L-glutamine and 10% fetal bovine serum (M.A. Bioproducts). Human non-small-cell lung carcinoma NSCLC-3 cells, established in culture (Wells *et al.*, 1994) from a surgical specimen was maintained in RPMI-1640 supplemented with 2 mM L-glutamine and 20% fetal bovine serum (FBS). Doubling time in culture of the HL-60 and NSCLC-3 cells was 40 h and 50 h respectively. The choice of these model systems was based on the need to establish the modulating efficacy of DPH with VBL in human tumours as well as tumours not restricted to the haematopoietic system.

The effect of DPH or OKA on the mitotic block and cytotoxicity induced by VBL was determined in HL-60 or NSCLC-3 cells treated for 48 h with DPH or OKA alone and in combination with VBL. Control and treated cells were subsequently analysed for: (a) cytotoxicity; (b) mitotic arrest and spindle damage by immunofluorescence using an α-tubulin antibody; and (c) apoptosis based on immunofluorescent detection of 3′-OH ends of DNA labelled with fluorescein-12-dUTP or DNA fragmentation by agarose gel electrophoresis.

### In vitro *cytotoxicity*

Cytotoxicity was determined either by trypan blue dye exclusion using a haemacytometer or in a soft-agar colony assay. The soft-agar colony assay was carried out by plating control or treated cells in RPMI-1640 supplemented with 2 mM L-glutamine, 20% FBS and 0.3% agar. Following incubation for 10 days in a humidified 5% carbon dioxide plus 95% air atmosphere, the colonies were counted using an automated counter (Ganapathi *et al.*, 1993). Colony forming efficiency under these conditions for control untreated HL-60 and NSCLC-3 cells was 14% and 7% respectively.

*Analysis of mitotic spindle morphology*

Cytospin preparations of control and treated cells were rapidly air dried and fixed for 20 min in 3.7% formaldehyde in a buffer containing 0.1 M 1,4-piperasine diethanesulfonic acid, pH 6.9, 1 mM magnesium sulphate and 2 mM ethyleneglycol bis (β-aminoethyl) ethyl -N,N,N',N'-tetraacetic acid - 2 M glycerol. Fixed cells were washed in phosphate-buffered saline (PBS), treated with 0.3% Nonidet P-40 in PBS, washed and stained with mouse monoclonal antibody to α-tubulin (Sigma, St Louis, MO, USA) for 30 min. Slides were air dried and stained with FITC or TRITC conjugated goat anti-mouse IgG F(ab')$_2$ antibody for 30 min (Cappel, Durham, NC, USA) and mounted in 1 mg ml$^{-1}$ *p*-phenylenediamine in 70% glycerol/PBS. Slides were examined in an Olympus BH2 microscope equipped with an epifluorescence attachment using: (a) exciter filter IF490 + EY455 and barrier filter Y455 for FITC; and (b) exciter filter IF-545 + BG-36 and barrier filter R610 for TRITC. The procedure described is an adaptation of that published by Leung *et al.* (1992). A minimum of 1000 cells in three separate fields were scored to determine the mitotic index. A minimum of 100 mitotic cells demonstrating multipolar spindle poles ($\geq 3$) were grouped to quantify abnormalities. Morphology of the spindles were also examined for collapse of the spindle and for heavily condensed and/or fragmented mitotic apparatus (Vandre and Wills, 1992).

*Detection of apoptotic cells*

Microscopic evaluation was carried out as described by Gavrieli *et al.* (1992). Briefly, cytospin preparations were labelled using terminal transferase and fluorescein-12-dUTP (Boehringer Mannheim, Indianapolis, Ins, USA). Slides were examined in a fluorescence microscope as described earlier and apoptotic cells scored by counting a minimum of 200 cells per field in three separate fields.

Agarose electrophoresis for internucleosomal fragmentation was carried out as described by Grant *et al.* (1992). Briefly, control and treated cells ($20 \times 10^6$) in lysis buffer (5 mM Tris base, 20 mM EDTA, 0.5% Triton X-100, pH 8.0 + 100 mg ml$^{-1}$ proteinase K) were incubated at 56°C for 18 h and centrifuged at 30 000 *g* for 45 min at 4°C to separate the low molecular weight fragments. The supernatant was subsequently incubated at 37°C for 4 h in the presence of 100 μg ml$^{-1}$ RNAase A. The electrophoresis was performed in 2% LMP agarose (BRL, Life Technologies, Gaithersburg, MD, USA) in TAE buffer (40 mM Tris-acetate, 1 mM EDTA, pH 8.3) containing ethidium bromide. Samples were loaded onto the gel after mixing with 15% Ficoll and a 100 bp DNA ladder was used as a marker. After overlay of the gel with TAE buffer electrophoresis was carried out for 3 h at 65 V and the gel visualised under ultraviolet light.

### Results and discussion

The data in Figure 1 demonstrate that DPH in a dose-dependent fashion enhances the cytotoxic effect of VBL in HL-60 or NSCLC-3 cells. While these observations extend our earlier findings with VCR, an important distinction was the requirement of simultaneous exposure to VBL plus DPH for at least one cell generation cycle for maximal potentiation of cytotoxicity. In order to determine a mechanistic basis governing the cellular effects of DPH in enhancing VBL cytotoxicity, HL-60 or NSCLC-3 cells were treated with VBL and/or DPH for 2 days followed by recovery for an additional 4 days in drug-free medium. The experimental design with HL-60 cells also involved high concentrations of VBL alone, to rule out that effects of VBL + DPH were merely related to altered drug levels and damage. The results in Figure 2a indicate that while differences between VBL or VBL + DPH were minimal during drug exposure, the recovery of proliferation in drug-free medium of VBL-treated cells was similar to the untreated control, in contrast to a persistent abrogation of proliferation in cells treated with VBL + DPH. The mitotic block induced (Figure 2b) between VBL or VBL + DPH was similar at the end of treatment and a rapid reversal to control values was apparent within 2 days in drug-free medium. In contrast, while apoptosis in VBL-treated cells during recovery in drug-free medium was similar to the control, the apoptotic response was maximal and persistent with VBL + DPH (Figure 2c). To characterise whether these findings with VBL + DPH were unique to leukaemic cells, the lung cancer NSCLC-3 model system was also evaluated. The data in Figure 3a demonstrate that only cells treated with VBL + DPH exhibit compromised recovery of proliferation in drug-free medium. The mechanistic basis for the compromised recovery of proliferation in drug-free medium in cells treated with VBL + DPH is possibly due to abnormal mitotic spindle (multipolar) morphology (Figure 3b) and the dominant persistence of an apoptotic response (Figure 3c). Overall, either in leukaemic HL-60 or lung cancer NSCLC-3 cells, the combination of VBL + DPH results in persistent growth arrest and apoptosis even in drug-free medium. Notably, within the range of VBL concentrations that can be achieved clinically with manageable toxicity (Rowinsky and Donehower, 1992), our data suggest that VBL, unlike VBL + DPH, is cytostatic. Data on double labelling (α-tubulin/fluorescein-12-dUTP) in Table I demonstrate that in HL-60 cells following treatment with VBL- + DPH, apoptotic cells were about 3-fold higher than cells arrested in mitosis. Since few if any cells were scored as mitotic and apoptotic, mechanistically our data demonstrate that the apoptosis induced by VBL + DPH is persistent even in interphase.

**Figure 1** Effect of DPH on VBL cytotoxicity in HL-60 (**a**) and NSCLC-3 (**b**) cells. Data are the mean value from at least duplicate experiments, s.d. < 15%. Survival of cells treated with 36.5 μM or 73 μM DPH alone was 97–100%. ■, 0 μM DPH; ▨, 36.5 μM DPH; ⬚ 73 μM DPH.

Based on the suggested role for protein phosphatases in egulating mitotic events and cytoskeletal integrity Yamashita et al., 1990; Eriksson et al., 1992; Fernande et ıl., 1992; Vandre and Wils, 1992; Gurland and Gundersen, 993), in the next series of experients using the HL-60 model ystem we investigated the effect of the protein phosphatase inhibitor OKA on the mitotic index, induction of apoptosis and cytotoxicity with VBL, using the human promyelocytic leukaemia HL-60 cells. The modulating effect of OKA on cytotoxicity was determined at the $IC_{50}$ of doxorubicin (DOX), VBL or taxol (TXL) alone, which was obtained from dose–response curves. The data in Figure 4 indicate that a concentration of 10 nM OKA, which by itself is marginally cytotoxic (<5% cell kill), enhances the cytotoxic effects of VBL >3-fold. In contrast, enhancement of cell kill observed with the combination of OKA and the mechanistically

**Figure 2** Proliferation (**a**), mitotic arrest (**b**) and apoptosis (**c**) in HL-60 cells following treatment with DPH, VBL or VBL + DPH for 2 days followed by recovery in drug-free medium for 4 days. Treatment was initiated at starting density of $0.5 \times 10^6$ cells $ml^{-1}$ and following treatment for 2 days samples were washed in drug-free medium, recovered by centrifugation and resuspended in fresh medium at a $0.3 \times 10^6$ trypan blue dye-excluding cells per ml. Control and treated samples were analysed as described in Materials and methods on days 2, 4 and 6.

**Figure 3** Proliferation (**a**), mitotic spindle pole number (**b**) and apoptosis (**c**) in NSCLC-3 cells following treatment with DPH, VBL or VBL + DPH for 2 days followed by recovery in drug-free medium for 4 days. Control and treated samples were analysed as described in Materials and methods on days 2, 4 and 6.

different microtubule poison, taxol or the topoisomerase II inhibitor doxorubicin is minimal. Statistical analysis of data in Figure 4 using a series of *t*-tests adjusted for multiple testing indicated that the combination of VBL + OKA was significantly ($P = 0.001$) more effective than either VBL or OKA alone. The results for TXL and DOX were less definitive. While the pleiotropic cellular effects of OKA are recognised, it is important to note that under the experimental conditions, OKA selectively enhances the cytotoxicity of VBL *vs* TXL or DOX, and the negative control compound, 1-nor-okadaone, which resembles OKA in physical properties and chemical structure, does not enhance VBL cytotoxicity at concentrations as high as 100 nM. The ability of OKA to mimic DPH in the potentiation of anti-mitotic effects and induction of apoptosis induced by VBL is also supported by data in Figure 5, which demonstrate that unlike VBL alone, the combination of VBL and OKA results in an increase of >3-fold and >2-fold of mitotic and apoptotic cells respectively.

Since cells demonstrating apoptosis based on labelling with fluorescein-12-dUTP exhibited nuclear chromatin condensation and segregation, we also determined internucleosomal DNA fragmentation by agarose gel electrophoresis. As shown in Figure 6 the formation of a 200 bp DNA ladder characteristic of apoptotic cell death is markedly enhanced only with the combination of VBL + OKA (Figure 6a) and VBL + DPH (Figure 6b).

Tumour cell insensitivity to VBL is generally accepted to be due to drug efflux by P-gp (Endicott and Ling, 1990; Gottesman and Pastan, 1993). We now demonstrate that the

**Table 1** Analysis of apoptosis and mitotic arrest in double labelled (α-tubulin/fluorescein-12-dUTP) HL-60 cells

| *Treatment*[a] | *Apoptotic index (%)*[b] | *Mitotic index (%)*[b] |
|---|---|---|
| Control | 2.8 ± 1.0 | 2.7 ± 1.3 |
| 73 μM DPH | 2.1 ± 1.0 | 2.8 ± 0.5 |
| 1.93 nM VBL | 12.6 ± 0.9 | 7.5 ± 0.9 |
| 1.93 nM VBL + 73 μM DPH | 31.2 ± 3.8 | 10.2 ± 2.3 |

[a]HL-60 cells were treated as indicated for 48 h and cytospin preparations stained with α-tubulin antibody and fluorescein-12-dUTP. [b]Slides were examined in a fluorescence microscope and a minimum of 200 cells per field in three separate fields scored. Values are mean ± standard deviation from a representative experiment. Few if any cells were identified to be in mitotic arrest and apoptotic.

**Figure 4** Modulation of vinblastine (VBL), taxol (TXL) and doxorubicin (DOX) cytotoxicity by 10 nM okadaic acid (OKA) or 100 nM 1-nor-okadaone (NOD) in human leukaemia HL-60 cells treated for 24 h with 2.75 nM VLB, 5 nM VLB, 5 nM TXL or 30 nM DOX. Concentrations of DOX, TXL or VBL producing about 50% cell kill were derived from dose-response curves and used to evaluate the effects of OKA or NOD on cytotoxicity. Cell survival was measured by a soft-agar colony assay. Range of values between replicate experiments was between 10% and 15%.

**Figure 5** Anti-mitotic and apoptotic response of HL-60 cells treated with 2.75 nM vinblastine (VLB) in the absence or presence of 10 nM okadaic acid (OKA). HL-60 cells were treated for 24 h and mitotic (**a**) or apoptotic (**b**) cells scored after staining with α-tubulin antibody or fluorescein-12-dUTP respectively.

**Figure 6** Agarose gel electrophoresis of DNA extracted from human promyelocytic leukaemia, HL-60 cells (**a**) or NSCLC-3 cells (**b**). HL-60 cells were treated for 24 h with: lane 1, 100 bp DNA ladder; lane 2, control; lane 3, 10 nM. OKA; lane 4, 2.75 nM vinblastine; and lane 5, 2.75 nM vinblastine plus 10 nM OKA. NSCLC-3 cells were treated for 48 h with: lane 1, control; lane 2, 73 μM DPH, lane 3, 1.93 nM VBL; lane 4, 1.93 nM VBL + 73 μM DPH; and lane 5, marker 100 bp DNA ladder.

anti-epileptic DPH (Rall and Schleifer, 1990) or the protein phosphatase inhibitor OKA (Vandre and Wills, 1992) can selectively enhance VBL cytotoxicity in human leukaemic (HL-60) or NSCLC-3 cells due to sustained growth arrest, damage to the mitotic spindle and induction of apoptosis. Both DPH or OKA can inhibit cell-cycle traverse in the mitotic cycle, leading to arrest in metaphase (MacKinney, 1980; Vandre and Wills, 1992). Metaphase arrest induced by DPH is suggested to involve effects on polymerisation of tubulin (MacKinney, 1978), while with OKA, inhibition of phosphatases involved in the mitotic cycle has been reported (Yamashita *et al.*, 1990; Vandre and Wills, 1992). Since concentrations of DPH that are capable of inducing measureable effects on polymerisation–depolymerisation kinetics of purified tubulin or metaphase arrest of treated cells are in excess of 300 μM (MacKinney, 1978), it is possible that alternate mechanisms are involved in the modulation of VBL cytotoxicity. Growth arrest by OKA in metaphase (Yamashita *et al.*, 1990; Vandre and Wills, 1992) is suggested to involve inhibition of dephosphorylation events regulating metaphase to anaphase transit (Yamashita *et al.*, 1990; Fernande *et al.*, 1992; Vandre and Wills, 1992). Other effects of OKA during the mitotic cycle include activation and inactivation of $p34^{cdc2}$ kinase and possible suppression of the cyclin proteolysis pathway (Yamashita *et al.*, 1990). There is no direct evidence demonstrating effects of OKA on tubulin, but inhibitors of protein phosphatases such as OKA and calyculin A affect regulation of microtubule stability by the selective breakdown of stable microtubules in fibroblasts or epithelial-like cells (Gurland and Gunderson, 1993).

The induction of apoptosis in OKA (>30 nM)-treated cells has been reported (Zheng *et al.*, 1994). While induction of apoptosis by DPH has not been reported, we have observed apoptosis only in cells treated with >300 μM DPH (data not shown). The results in Figures 2, 3, 5 and 6 demonstrate that 73 μM DPH or 10 nM OKA alone has a minimal effect on apoptosis, but in combination with VBL pronounced and persistent apoptosis is observed. Notably, enhanced cytotoxicity with VBL plus DPH or OKA may be related to apoptotis during interphase, since cells labelled with fluorescein-12-dUTP and identified as apoptotic were not arrested in mitosis. Also, while growth arrest of VBL-treated cells is apparent only during treatment, cells treated with VBL plus DPH or OKA exhibit continued growth arrest in drug-free medium. Thus the potentiation of VBL cytotoxicity by DPH or OKA is due to prolonged inhibition of proliferation and consequent apoptosis. Apoptosis in response to cellular damage is dependent on the expression of WT p53 (Oren, 1994). The pronounced apoptosis observed in this study with HL-60 or NSCLC-3 cells treated with VBL + DPH is thus of interest since: (a) HL-60 cells do not express p53 due to extensive deletion of the gene (Wolf and Rotter, 1985; Collins 1987); and (b) NSCLC-3 cells are hemizygous for p53 expression based on the absence of a WT allele and expression of mutant p53 with a tyrosine→cysteine mutation at amino acid 163 (R Ganapathi *et al.*, manuscript in preparation). Ongoing studies demonstrate that the modulating effects of DPH or OKA on VBL cytotoxicity is also observed in HL-60 or NSCLC-3 cells with the MDR phenotype (R Ganapathi *et al.*, manuscript in preparation). Since neither DPH or OKA bind to P-gp (Chambers *et al.*, 1993; Ganapathi *et al.*, 1993) the differential effects of OKA or DPH on MDR-associated drugs (VBL *vs* TXL or DOX) is novel, since increasing drug accumulation by interfering with P-gp function is the strategy for enhancing drug cytotoxicity (Georges *et al.*, 1990). The potential therapeutic benefit with the combination of DPH and VBL in a clinical setting is currently under investigation in a phase I setting, and preliminary results suggest that DPH (400 mg $day^{-1}$) can be safely administered in combination with 2.25 mg VBL $m^{-2}$ $day^{-1}$ as a continuous infusion for 5 days (Peereboom *et al.*, 1995).

Overall, our results demonstrate that the protein phosphatase inhibitor OKA can mimic the cellular effects of DPH in selectively enhancing the cytotoxicity of VBL. Since under the experimental conditions evaluated VBL is cytotstatic *vs* VBL plus DPH or OKA cytocidal, interfering with metaphase transit, damage to the mitotic spindle and apoptosis during interphase are mechanistically involved in the potentiation of VBL cytotoxicity. These novel results suggest the use of DPH or OKA as a powerful tool to study cellular effects of vinca alkaloids and possibly for the development of novel therapeutic strategies.

### Abbreviations

VCR, vincristine; VBL, vinblastine; DPH, phenytoin; OKA, okadaic acid; DOX, doxorubicin; TXL, taxol; FBS, fetal bovine serum; P-gp, P-glycoprotein; PBS, phosphate-buffered saline.

### Acknowledgements

The authors acknowledge Sharon VanderBrug Medendorp, MPH, for statistical analysis of the data; Audrey Brickenden for her excellent secretarial assistance; and Jim Reed of the Art-Medical Illustrations and Photography Department for skilful preparation of the art work. This work was supported by Public Health Service Grant RO1 CA35531, awarded by the National Cancer Institute, Department of Health and Human Services.

### References

CHAMBERS TC, RAYMOR RL AND KUO JF. (1993). Multidrug-resistant human KB carcinoma cells are highly resistant to the protein phosphatase inhibitors okadaic acid and calyculin A. Analysis of potential mechanisms involved in toxin resistance. *Int. J. Cancer*, **53**, 323–327.

COLLINS SJ. (1987). The HL-60 promyelocytic leukemia cell line: Proliferation, differentiation and cellular oncogene expression. *Blood*, **70**, 1233–1244.

ENDICOTT JA AND LING V. (1989). The biochemistry of P-glycoprotein-mediated multidrug resistance. *Annu. Rev. Biochem.*, **58**, 137–171.

ERIKSSON JE, BRAUTIGAN DL, VALLEE R, OLMSTED J, FUJIKI H AND GOLDMAN RD. (1992). Cytoskeletal integrity in interphase cells requires protein phosphatase activity. *Proc. Natl Acad. Sci. USA*, **89**, 11093–11097.

FERNANDE A, BRAUTIGAN DL AND LAMB NJC. (1992). Protein phosphatase type 1 in mammalian cell mitosis chromosomal localization and involvement in mitotic exit. *J. Cell Biol.*, **116**, 1421–1430.

GANAPATHI R, HERCBERGS A, GRABOWSKI D AND FORD J. (1993). Selective enhancement of vincristine cytotoxicity in multidrug-resistant tumor cells by Dilantin (Phenytoin). *Cancer Res.*, **53**, 3262–3265.

GAVRIELI Y, SHERMAN Y AND BEN-SASSON SA. (1992). Identification of programmed cell death *in situ* via specific labeling of nuclear DNA fragmentation. *J. Cell Biol.*, **119**, 493–501.

GEORGES E, SHAROM F AND LING V.(1990). Multidrug resistance and chemosensitization: Therapeutic implications for cancer chemotherapy. *Adv. Pharmacol.*, **21**, 185–220.

GOTTESMAN MM AND PASTAN I. (1993). Biochemistry of multidrug reistance by the multidrug transporter. *Annu. Rev. Biochem.*, **62**, 385–427.

GRANT S, JARVIS WD, SWERDLOW PS, TURNER AJ, TAYLOR RS, WALLACE HJ, LIN P-S, PETTIT GR AND GEWIRTZ DA. (1992). Potentiation of the activity of 1-β-D-arabinofuranosylcytosine by the protein kinase C activator bryostatin 1 in HL-60 cells: association with enhanced fragmentation of mature DNA. *Cancer Res.*, **52**, 6270–6278.

GURLAND G AND GUNDERSEN GG. (1993). Protein phosphatase inhibitors induce the selective breakdown of stable microtubules in fibroblasts and epithelial cells. *Proc. Natl Acad. Sci, USA*, **90**, 8827–8831.

KERR JFR, WINTERFORD CM AND HARMON BV. (1994). Apoptotis: Its significance in cancer and cancer therapy. *Cancer*, **73**, 2013–2026.

LEUNG MF, SOKOLOSKI JA AND SARTORELLI AC. (1992). Changes in microtubules, microtubule associated proteins, and intermediate filaments during differentiation of HL-60 leukemia cells. *Cancer Res.*, **52,** 949-954.

MACKINNEY AA, VYAS RS AND WALKER D. (1978). Hydantoin drugs inhibit polymerization of pure microtubular protein. *J. Pharmacol. Exp. Ther.*, **204,** 189-194.

MACKINNEY AA, VYAS R, MUELLER C AND GORDER CA. (1980). Comparison of potency of hydantoins in metaphase arrest and inhibition of microtubular polymerization. *Mol. Pharmacol.*, **17,** 275-278.

OREN M. (1994). Relationship of p53 to the control of apoptotic cell death. *Semin. Cancer Biol.*, **5,** 221-227.

PEEREBOOM DM, GANAPATHI R, AARON R, MCLAIN DA, BUDD GT, OLENCKI TE AND BUKOWSKI RM. (1995). Modulation of resistance to vinca alkaloids: a phase I trial of vinblastine with phenytoin. *Proc. Am. Assoc. Cancer Res.*, **36,** 246.

RALL TW AND SCHLEIFER LS. (1990). Drugs effective in the therapy of the epilepsies. In *The Pharmacological Basis of Therapeutics*, Gilman AG, Rall TW, Nies AS, Taylor P (eds). pp. 436-462, Pergamon Press: New York.

ROWINSKY EK AND DONEHOWER RC. (1992). Vinca alkaloids and epipodophyllotoxins. In *The Chemothrapy Source book*, Perry MC (ed). pp. 359-383, Williams and Wilkins: Philadelphia.

SMETS CA. (1994). Programmed cell death (apoptosis) and response to anti-cancer drugs. *Anti Cancer Drugs*, **5,** 3-9.

VANDRE DD AND WILLS VL. (1992). Inhibition of mitosis by okadaic acid: possible involvement of a protein phosphatase 2A in the transition from metaphase to anaphase. *J. Cell Sci.*, **101,** 79-91.

WELLS NJ, ADDISON CM, FRY AM, GANAPATHI R AND HICKSON ID. (1994). Serine 1524 is a major site of phosphorylation on human topoisomerase IIα protein *in vivo* and is a substrate for casein kinase II *in vitro*. *J. Biol. Chem.*, **209,** 29746-29751.

WOLF D AND ROTTER V (1985). Major deletions in the gene encoding the p53 tumour antigen cause lack of p53 expression in HL-60 cells. *Proc. Natl Acad. Sci. USA*, **82,** 790-794.

YAMASHITA K, YASUDA H, PINES J, YASUMOTO K, NISHITANI H, OHTSUBO M, HUNTER T, SUGIMURA T AND NISHIMOTO T. (1990). Okadaic acid, a potent inhibitor of type 1 and type 2A protein phosphatases, activates cdc2/H1 kinase and transiently induces a premature mitosis-like state in BHK21 cells. *EMBO J.*, **9,** 4331-4338.

ZHENG B, CHAMBERS TC, RAYNOR RL, MARKHAM PN, GEBEL HM, VOGLER WR AND KUO JF. (1994). Human leukemia K562 mutant (K562/OA200) selected for resistance to okadaic acid (protein phosphatase inhibitor) lacks protein kinase, exhibits multidrug resistance phenotype and exhibits drug pump P-glycoprotein. *J. Biol. Chem.*, **269,** 12332-12338.